# Photovoltaic Panels Classification Using Isolated and Transfer Learned Deep Neural Models Using Infrared Thermographic Images

**DOI:** 10.3390/s21165668

**Published:** 2021-08-23

**Authors:** Waqas Ahmed, Aamir Hanif, Karam Dad Kallu, Abbas Z. Kouzani, Muhammad Umair Ali, Amad Zafar

**Affiliations:** 1 Department of Electrical Engineering, University of Wah, Wah Cantt 47040, Pakistan; waqas.ahmed.ee@wecuw.edu.pk (W.A.); dr.aamirhanif@wecuw.edu.pk (A.H.); 2 Department of Robotics and Intelligent Machine Engineering (RIME), School of Mechanical and Manufacturing Engineering (SMME), National University of Sciences and Technology (NUST) H-12, Islamabad 44000, Pakistan; karamdad.kallu@smme.nust.edu.pk; 3 School of Engineering, Deakin University, Geelong, VIC 3216, Australia; kouzani@deakin.edu.au; 4 Department of Unmanned Vehicle Engineering, Sejong University, Seoul 05006, Korea; 5 Department of Electrical Engineering, Islamabad Campus, University of Lahore, Islamabad 54590, Pakistan

**Keywords:** deep convolution neural network, PV panels, infrared images, hotspots

## Abstract

Defective PV panels reduce the efficiency of the whole PV string, causing loss of investment by decreasing its efficiency and lifetime. In this study, firstly, an isolated convolution neural model (ICNM) was prepared from scratch to classify the infrared images of PV panels based on their health, i.e., healthy, hotspot, and faulty. The ICNM occupies the least memory, and it also has the simplest architecture, lowest execution time, and an accuracy of 96% compared to transfer learned pre-trained ShuffleNet, GoogleNet, and SqueezeNet models. Afterward, ICNM, based on its advantages, is reused through transfer learning to classify the defects of PV panels into five classes, i.e., bird drop, single, patchwork, horizontally aligned string, and block with 97.62% testing accuracy. This proposed approach can identify and classify the PV panels based on their health and defects faster with high accuracy and occupies the least amount of the system’s memory, resulting in savings in the PV investment.

## 1. Introduction

Electrical energy fulfils the human demand in every sector, i.e., industry, commercial, agriculture, education, etc.; therefore, it has an ever-growing demand [1,2]. However, the majority of the energy production sources across the globe are fossil fuel-based power plants, even though fossil fuels are limited in nature [1,2,3]. For example, Pakistan is heavily dependent upon conventional thermal power plants for its energy needs [3,4]. Moreover, the burning of fossil fuels such as oil, gas, coal, etc., emits greenhouse gases such as carbon dioxide, methane, nitrous oxide, water vapours, etc., which trap solar radiation in the earth’s atmosphere and cause significant environmental degradation such as climate change [2,3,5,6]. As per the literature, electrical energy generation and consumption account for two-thirds of global stack emissions [7].

To achieve green energy sustainability, solar energy, a green, low carbon renewable energy source, is one of the best energy production alternatives with 26–217 gCO2eq/kWh emissions compared to the 530 gCO2eq/kWh emissions of thermal plants on a global average [2]. As per the literature, solar photovoltaics (PV) along with wind energy sources are expected to meet 88% of global energy demand by 2050 [1]. However, investment in a PV system is high, and the payback time defines PV system economic viability, which is highly dependent upon PV system performance and lifetime [3,8]. Therefore, PV system manufacturers must guarantee their long-term performance and lifespan, which is reflected in the PV datasheet [9]. PV system performance suffers with time due to aging, which is a loss process, or due to defects such as component breakdown, which has an immediate impact [10]. Moreover, in the case of PV system defects, PV efficiency and output suffer from a noticeable impact on payback time [3,8].

### 1.1. Losses in PV System

The power loss in a PV system arises due to different factors, including manufacturing defects, transportation, installation, short circuit, open circuit, partial shading, shading, line to line fault, arc fault, bird drops, dust accommodation, module mismatch, environmental degradation, ageing, cell cracking due to mechanical stresses, etc., in real-time operations [1,8,11,12,13,14,15,16,17]. Details on PV system defects are provided in Basnet et al. [18]. These defects may limit 50% of power output per module [11]. Generally, all defects are treated as faults in the system. However, defects such as bird drops, shadows, dust accommodation, etc., reduce the PV output by localised heating of the PV cell (hotspot formation) due to the operation in the reverse region and dissipating healthy cell current due to series connection [1,14,19]. If such issues remain undetected and persist on the PV surface, these issues result in permanent failure/faults of PV panels [20]. Moreover, defects/hotspots formation on PV panels’ occurrence frequency, duration of stay, and intensity are unpredictable and are purely natural parameters [21]. In addition, only a few defects are identifiable through naked eye/visual inspection, such as corrosion, burned and broken cells, bubbles, etc. [19]. Therefore, a fast, low-storage, and accurate identification system will ensure the safe operation of the PV system during its lifetime by the timely identification of hotspots/defects/faults in the PV system during its normal operation [22]. However, it is important that if defects do not affect the output or system safety, the defect is not treated as a failure [8]. 

### 1.2. Health Monitoring Approaches of PV System 

To prolong the performance of PV panels, an accurate and efficient approach to identify and classify PV panels because of defects is inevitable [1,11]. Generally, PV system health and defects are identified using two broad approaches: through electrical signal (voltage and current characteristics of PV panels) [16,17,18] and non-invasive image-based approaches, such as electroluminescence (EL) images [23], infrared (IR) thermography of PV panels [1,8,11,12,19], fluorescence images [24], photoluminescence images [25] etc. However, IR thermography of PV panels is widely used in the literature due to its speed, low cost, large-scale outdoor applications, user friendliness, and accuracy [8,11,12,19].

### 1.3. Non-Invasive Images-Based Classification of PV Panels’ Health and Defects: Literature Review

Different image processing-based machine learning and deep learning approaches are utilised to identify and classify the PV system defects based on images. Niazi et al. [26] used the naïve-Bayes approach to classify PV panels into two classes, defective and non-defective, with IR thermograph texture features with a 98.4% mean recognition rate. Meanwhile, Ali et al. [1] used a support vector machine on IR images after extracting image features and classified PV panels into three classes based on health with 92% accuracy. Naïve Bayes was also used to classify PV panels into three categories (defective, non-defective with hotspots, and non-defective without hotspots) using an IR thermograph dataset with a 94.1% mean recognition rate [27]. 

However, in deep learning, i.e., using neural networks, Dunderdale et al. [11] used IR images to classify PV panels into defective and non-defective with 91.2% accuracy and defects (block, patchwork, single, and string) identification with 89.5% accuracy using a scale-invariant feature transform feature descriptor, spatial pyramid matching, and deep learning approaches. Akram et al. [12] developed an isolated light convolution isolated neural network trained on PV system EL thermographs, and they used a transfer learning approach on PV system IR thermography for defect detection with 99.23% accuracy. Similarly, the isolated convolution neural networks to classify IR images based on dust and hotspots with 98% accuracy were proposed by Cipriani et al. [13]. Kurukuru et al. [28] used IR thermographs of eight different panels (one healthy and seven faulty), extracted texture features, and used a scaled conjugate gradient back propagation algorithm to adjust the weight of the neural network classifier and achieved 91.7% testing accuracy. Bommes et al. [29] used aerial IR videos of PV modules and a pre-trained convolutional neural network, ResNet-50, to classify the 10 common module abnormalities with more than 90% testing accuracy. 

The PV systems have received worldwide acceptance as a green energy solution. However, the defects/hotspots/faults in the PV systems that arise due to the environment, manufacturing, transportation, operation, etc., greatly reduce their efficiency and performance. In this study, an isolated convolution neural model (ICNM) was built from scratch to classify PV panels based on their health into three categories—healthy, hotspot, and faulty—using IR images. The hotspot PV class is the class suffering from dust, shadows, bird drop issues, etc., and a timely solution to these issues may revert their state to first class or healthy. Afterward, the results of the isolated model are compared with transfer learned pre-trained networks such as ShuffleNet, GoogleNet, and SqueezeNet in terms of storage space through their architecture complexity, fastness, true positive rates (TPR), false negative rates (FNR), positive predictive values (PPV), false discovery rate (FDR), and accuracy. Finally, ICNM advantages are utilised using the transfer learning approach to classify five defects in PV panels—namely, bird drops, block, single, patchwork, and horizontally aligned (HA) string—which arise due to different environmental issues and were initially adjusted as two broad health classes, i.e., hotspot and faulty through neurons weights. 

The rest of the study is structured as follows. Section 2 explains the research approach. Section 3 presents the results followed by discussion, Section 4 and finally, the study conclusion is provided in Section 5.

## 2. Research Approach

**Figure 1:**
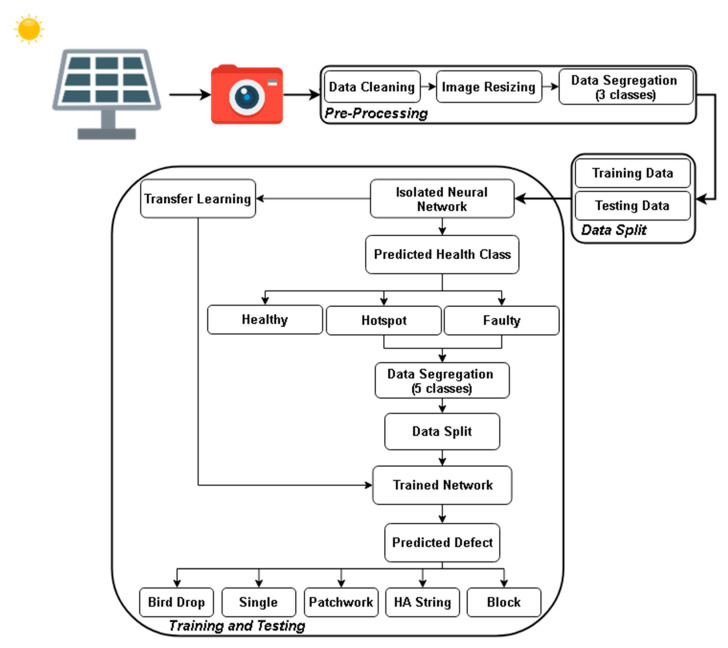
Proposed approach.

This study utilises an IR image dataset of a 42.24 kW solar photovoltaic system located in Lahore, Pakistan [27]. The acquired images are pre-processed for uniformity by removing unwanted noise. Afterward, the dataset is first segregated into sub-classes, i.e., based on their health, i.e., healthy, hotspot, and faulty. Secondly, data of two classes, i.e., hotspot and faulty, are further segregated into five sub-classes of defects, i.e., bird drops, single, block, patchwork, and HA string. After segregation, pre-processed images are split into training data comprised of 80% randomly selected images and 20% testing data comprised of the remaining images. Both training and testing datasets are created with an equal proportion of images to train the networks properly. A validation dataset is a 20% dataset created to test the models’ design to avoid over-fitting and a performance check of models by further splitting the training dataset. Finally, a three-class ICNM is built, trained, and validated against transfer-learned pre-trained networks, i.e., 50-layered ShuffleNet, 22-layered GoogleNet, and 18-layered SqueezeNet. Afterward, the advantages of ICNM, fast response, simple architecture, and accuracy are used through a transfer learning approach to classify the five different defects in PV panels. The proposed approach is presented in Figure 1. The study was carried out on MATLAB 2020a with the following system specifications: i5 7th generation, 16 GB DDR4 RAM with a 500 GB SSD hard disk, 2GB Ati Radeon Graphics Card, 64-bit operating system, and x64-based processor.

### 2.1. PV System

PV system output and performance vary due to multiple parameters such as geographical parameters, i.e., ambient temperature, precipitation, daily solar radiation, etc., and orientation angles [2]. For this study, a previously published PV dataset [27], a PV system installed in Lahore, Pakistan, was obtained and used to classify PV panels based on health and defects to restrain power losses. The per annum average of geographical parameters for Lahore city is provided in Table 1. The data is retrieved from the NASA Meteorological Database using RETScreen Expert software [30]. 

The literature suggests that bodies with a temperature above absolute zero, zero kelvin, possess thermodynamic energy and emit electromagnetic radiation in the infrared region, with a wavelength of 8 to 12 mm [1]. However, some literature suggests that wavelengths are 700 nm to 1 mm with a 430 THz to 300 GHz frequency and 1.7 eV to 1.24 meV photonic energy [31]. Infrared thermography was used to examine the PV system’s health, heat dissipation, and defects based on the thermal properties. The infrared camera, PV system, and on-site geographical parameters are provided in Table 2. Details of the experimental setup are provided in Niazi et al. [27].

### 2.2. Pre-Processing

**Figure 2:**
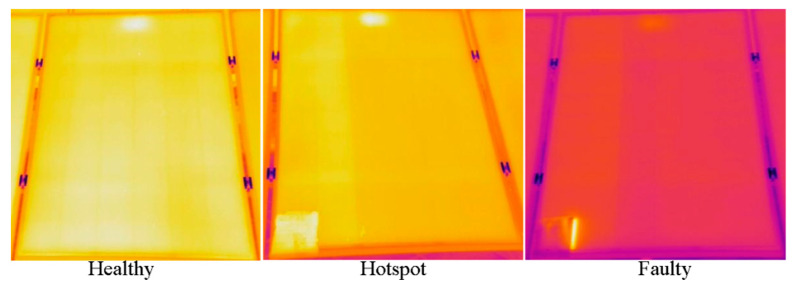
Solar panels classification based on health.

Raw infrared image datasets of 42.24 kW PV systems are pre-processed to increase classification accuracy and reduce unwanted mismatches. Firstly, PV system datasets were cleaned by removing unwanted background objects such as ground, trees, railings, etc., as much as possible. Afterward, images were resized to obtain uniformity. A dataset of 315 infrared images based on PV system health was used. For each sub-class, data is provided in Table 3 and images are reflected in Figure 2. 

**Figure 3:**
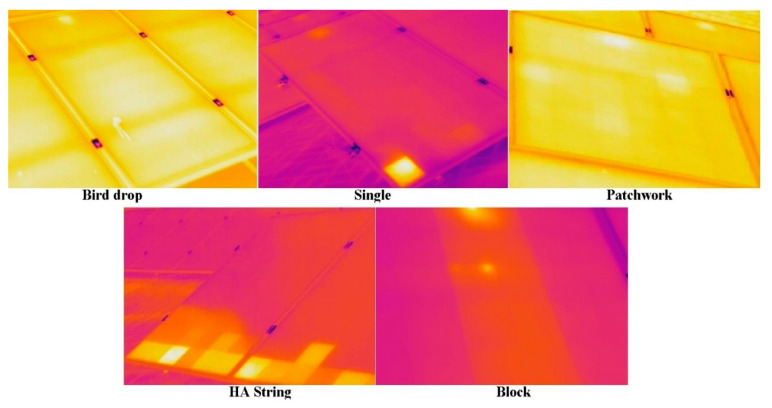
Solar panels classification based on defects.

Moreover, different PV defects on inspection of a thermal image are easily identifiable [11]. However, it is not possible for a large-scale PV system scenario. However, in defects, birds drop defects that have no specific shape and location, as they are highly unpredictable. The single defect is identifiable as a small rectangular shape. A patchwork defect consists of multiple single defects scattered on the PV surface. The HA string defect is a single defect in a row. Moreover, block defects almost cover up to one-third of PV panels. All these defects are visible due to hotter regions compared to the rest of the PV panels, as shown in Figure 3. Extensive details are provided in [11,32]. A dataset of 213 infrared images for defects on PV panels is provided in Table 4.

### 2.3. Isolated and Trained Transfer Learning-Model-Based Classifiers 

The classifiers are rule-based systems capable of data transfer with a parallel processing pool. Classifiers continuously receive new information and process it based on capabilities already learned [33]. In this era, deep neural network models are one of the most powerful tools utilised for classification [23]. Based on deep neural network classifiers, there are mainly two approaches: isolated neural network and transfer learning [12]. 

In an isolated neural network model, first, a new model is prepared and trained from scratch without any previous knowledge, without adjusting the weights of its neurons to any other problem [12]. In the transfer learning approach, a neural network model has been trained on a base image dataset for a given problem; the model-learned knowledge, i.e., neurons’ weight adjustment, layers, and connections (architecture) advantages, are reutilized, or learning is transferred to a new and different image dataset. The architecture of models concisely trained on different classification problems through transfer learning is reused for new problem solutions [12]. The advantage transfer learning offers is that its architecture, i.e., layers, connections, and weight adjustment, is reutilized for a new problem solution rather than building a new model from scratch that is trained iteratively for the desired performance, which results in fast convergence and less data for training. Two main approaches to transfer learning are pre-trained model-based transfer learning and a model that is built from scratch, trained, and afterwards utilize the transfer learning approach for a new issue [12,34]. However, pre-trained deep networks are publicly available, such as GoogleNet [35], SqueezeNet [36], Resnet50 [23,37], MobileNet v2 [11,23], Inception V3 [23], ShuffleNet [38], etc., and are widely used for classification problems. 

**Figure 4:**
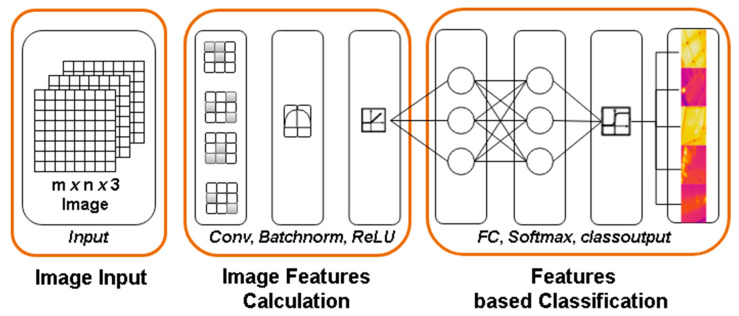
Deep neural network generic architecture for PV classification.

The generic architecture of an ICNM for features extraction and classification of input images based on features is provided in Figure 4. In this study, IR images were used to classify PV panels based on their health. For this purpose, different architectures of ICNM are created from scratch, trained, and validated to avoid over-fitting. Secondly, pre-trained deep neural networks, i.e., SqueezeNet, GoogleNet, and ShuffleNet, after the transfer learning approach, are utilised on the same dataset to validate the performance of ICNMs in terms of storage space due to architecture complexity, TPR, FNR, PPV, FDR, execution time, and accuracy. Finally, the best-isolated neural model is reutilised by the transfer learning approach to re-adjust the neurons’ weights to classify the five different defects of PV panels into their sub-classes, which were initially classified as hotspots or faulty classes. 

### 2.4. Training and Testing Dataset

Pre-processed IR image datasets are divided into training and testing data. The training dataset is formed by randomly distributing 80% of the images, which are provided in Table 3 and Table 4, into new datasets to train the deep neural networks. The remaining 20% of the image datasets are utilised to test the efficiency and accuracy of the models. Moreover, each dataset is split into both categories randomly and in equal proportion to ensure the proper training and testing of the deep neural network models. 

## 3. Results

Training and validation parameters for all schemes were kept constant for comparison, provided in Table 5.

### 3.1. Isolated Neural Network and Transfer Learned Pre-Trained Networks for PV Classification Based on Health

**Figure 5:**
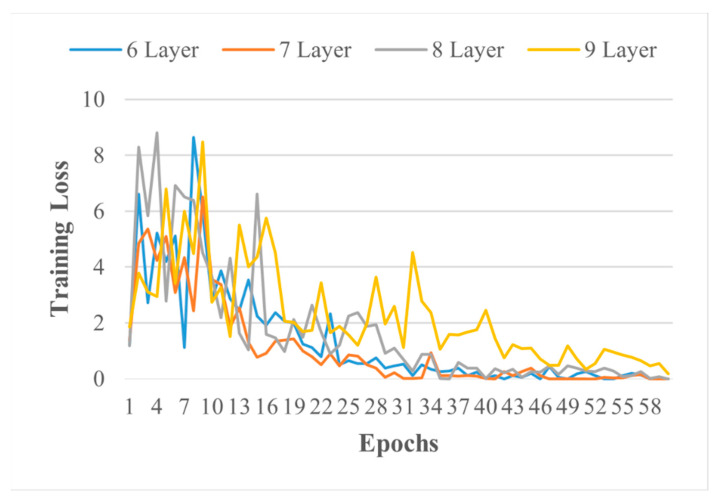
Training loss of different-layered ICNMs.

**Figure 6:**
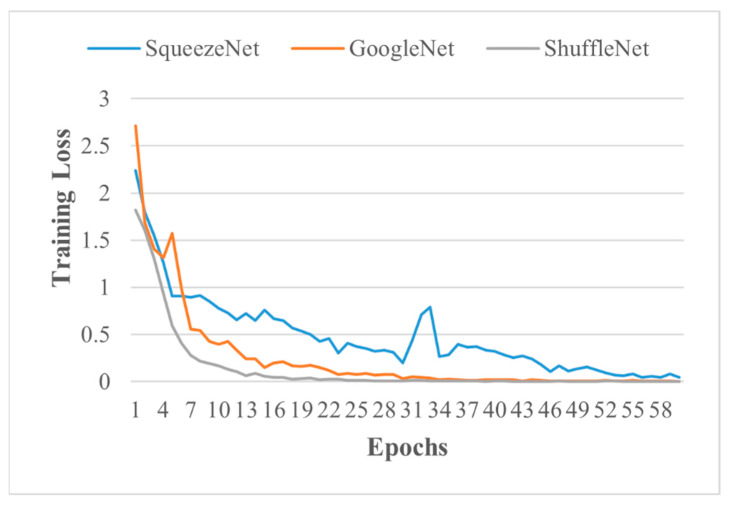
Training loss of pre-trained networks.

Firstly, the classification of PV panels based on their health is conducted using ICNMs. Different architectures of the ICNM were built by varying convolution layers, with batch normalisation, with and without activation functions, etc. ICNM based on higher validation accuracy, TPR, FNR, PPV, FDR, and validation loss is selected and compared with transfer learned, SqueezeNet, GoogleNet, and ShuffleNet, pre-trained networks from open sources, MathWorks. Detailed comparisons of models are provided based on training loss, training accuracy, validation loss, validation accuracy, execution time, and architecture complexity. Table 6 provides the information of ICNMs, whereas Table 7 reports the information on the pre-trained networks. All models were validated on the IR images dataset that was not utilised in the training phase. Figure 5 and Figure 6 show the training loss of different layer ICN models and a pre-trained network for health classification of the PV system over 60 epochs, respectively. Training loss is provided on the vertical axis, and the epochs/experience are provided on horizontal axis. Training loss diagnoses the condition of model; for instance, six and seven-layered ICNMs loss is minimising, which represents their good learning compared to eight and nine-layered ICNMs and pre-trained networks. Moreover, seven-layered ICNMs model training loss reaches stable points with the least variations, which means good fit learning.

### 3.2. Transfer Learning on an Isolated Model for Defects Classification

**Figure 7:**
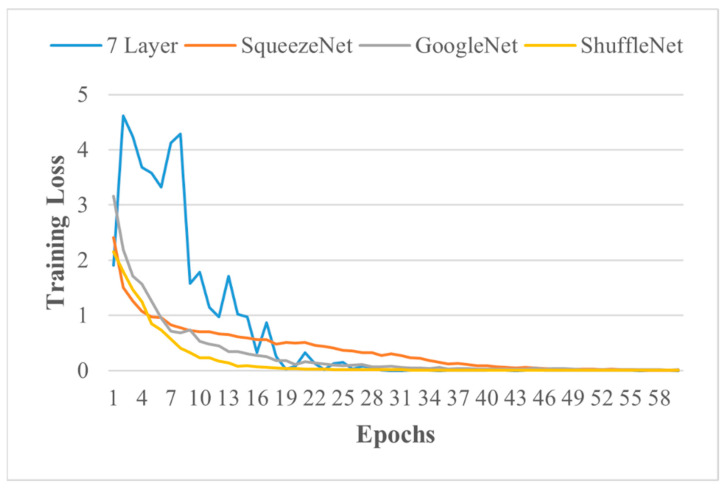
Training loss of models.

The seven-layered ICN model initially trained on IR images to classify PV panels into three classes based on health was re-utilised after the transfer learning approach to classify PV panels defects among two health sub-classes, i.e., hotspot and faulty, due to its training accuracy, training loss, validation loss, validation accuracy, execution time, simple architecture, and lower storage requirement. The testing accuracy of the seven-layered transfer learned model for five defects is provided in Table 8, while training loss is illustrated in Figure 7. Pre-trained network results on five defects classification are provided in Table 9. It is noted that TPR, FNR, PPV, and FDR are based on testing results on trained networks.

### 3.3. Transfer Learning on Augmented Dataset

**Figure 8:**
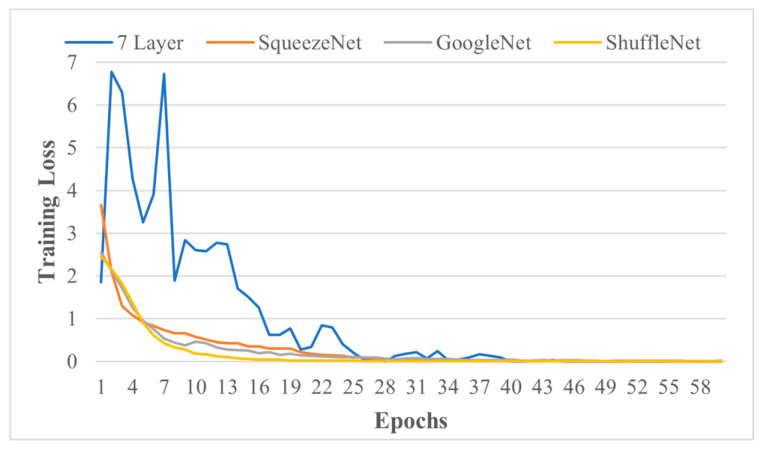
Training loss of models for augmented datasets.

The defects-based dataset is imbalanced since defects occurrence and frequency is a natural parameter [21]. A new balanced augmented dataset of defects based on 275 images (with 20% of each class) is created using scaling and shifting (0–20°) with noise [39]. Afterwards, data are split randomly in an 80:20 training and testing ratio but with equal proportions, and models are trained. The training loss of the model with augmented datasets of defects is provided in Figure 8, and detailed results of the models’ performance are provided in Table 10. TPR, FNR, PPV, and FDR are based on testing accuracy. MATLAB 2020a with core™ i5, 8 GB Ram was used for this section. 

## 4. Discussion

PV systems are widely used globally because of their green renewable energy nature, while defects blocking the PV cell’s radiation absorption capability result in current dissipation, localised heating, and in turn energy and investment loss. Defects that remain undetected lead to the total faultiness of PV panels. Therefore, different methods are used to monitor the condition of PV panels/modules for prediction-based maintenance. For predicting maintenance, different approaches such as electroluminescence, thermographs, UV-fluorescence, transmission methods, etc. are used [40]. Therefore, in this study, IR thermographs are carried out for prediction-based maintenance.

In the literature, linear and nonlinear, feature-based, deep network-based classifiers, etc. are extensively used for PV system classification [1,11,12,13,26,27,28,29]. Linear and feature-based classifiers such as SVM, naïve Bayes, etc., are used to differentiate PV panels based on their health or defects into two and three categories. These classifiers have the advantage of simple structure, less complexity, storage requirement, and execution time. Moreover, their accuracy increases with the most appropriate feature selection in the dataset. In contrast, nonlinear and deep network-based classifiers such as convolution neural networks offer an advantage in terms of multi-classes-based classification because of their complex architecture and nonlinearity, resulting in higher accuracy. These classifiers extract the most relevant features from the dataset and adjust the neuron’s weight iteratively to classify multiple defects.

However, linear and feature-based classifier accuracy suffers from increased output classes because of their simple architecture. Moreover, their accuracy is highly dependent upon the most relevant feature extraction. The image’s dataset in different environmental conditions may require different features, resulting in further limitations of these classifiers. On the other hand, nonlinear and deep network-based classifiers, due to their complex architecture, need more storage and advanced computing systems for their long execution time, even in fewer classes-based differentiation problems. 

In this study, multi-classes-based classification with less execution time, high accuracy, less architectural complexity, and storage requirements issues are addressed. Firstly, the IR images dataset was split into three health-based categories using a simple seven-layered ICNM with 96% accuracy. Afterward, isolated trained ICNM was re-utilised using the transfer learning approach to classify the five defects of the PV system with 97.63% testing accuracy in eight minutes. Moreover, a new augmented dataset of five-class defects was created using scale and shift, with associated noise to balance the dataset. A seven-layer transfer learned model and pre-trained networks were re-trained, validated, and tested on an augmented images dataset. The seven-layered transfer learned model has the highest validation accuracy, with a testing accuracy of 96.36% in the presence of a noisy augmented dataset. The seven-layer transfer learned model has the advantage of the least computational time requirement, storage requirement, architecture complexity, and computing system requirements compared to pre-trained networks. It is important that ICNM 7-layered includes input and output not only hidden layers.

Moreover, the direct classification of a PV system into six classes based on health and five defects requires modifications in ICNM to extract its most relevant features, which increases the complexity, storage requirement, and execution time. However, in this approach, initially, three health-based classes adjusted the neurons’ weights in eight minutes, and through transfer learning, a seven-layered ICNM trained model after neurons weight re-adjustment classified five defects with higher accuracy in less time compared to other pre-trained networks with no increase in architectural complexity and through a simple computing system. 

## 5. Conclusions

PV panels are widely used across the globe as a promising solution to global climate change, introducing reliability, investment, and output loss in the system due to hotspots and faultiness. The timely monitoring of PV panel health and defects can avoid their permanent failure. In this paper, a fast, low-storage, and simple architecture of an isolated deep convolution-based neural model was built and utilised with 96% accuracy to classify PV panels into three categories based on health. Afterward, a transfer learning approach was utilised on the same isolated built model to classify the PV panels’ defects, initially placed in hotspot, and faulty with 97.62% testing accuracy on a new IR images dataset not used in training and validation in eight minutes. However, an augmented images dataset of defects resulted in 96.36% testing accuracy. The isolated neural network results are validated against pre-trained high storage, complex architecture, and high execution time neural networks. This approach can classify PV panels to restrain the power loss and ensure the shortest payback time possible. Future work includes the testing of ICN models on big datasets without the need of data augmentation. 

## Figures and Tables

**Table 1:** Geographical parameters of Lahore city, Pakistan.

Parameter	Values
Geographical coordinates	31.5 N, 74.4 E
Air temperature	24.4 °C
Relative humidity	61.6%
Precipitation	551.78 mm
Daily solar radiation—horizontal	4.68 kWh/m^2^/d
Wind speed at 10 m	2.1 m/s

**Table 2:** Details of experimental setup.

Parameter	Values
PV system	42.24 kW
PV strings	8
PV modules per string	22
PV panel rating	240 W
Thermal camera	FLIR VUE-Pro 640
Thermal camera position	Handheld, horizontal aligned
Ambient temperature	32–40 °C
Wind speed	6.9 m/s
Irradiance level	700 W/m2
Thermal image- bit depth	8-bit
Spatial resolution	640 × 512/pixel

**Table 3:** Classification of IR images dataset based on health condition.

PV System Health	Images Set
Healthy	32.38%
Hotspot	31.43%
Faulty	36.19%

**Table 4:** Details of experimental setup.

PV System Health	Images Set
Bird Drop	5.16%
Single	36.62%
Patchwork	5.16%
HA String	12.68%
Block	40.38%

**Table 5:** Training parameters used in deep convolution neural networks.

Solver	SGDM
Initial learn rate	0.001
Epochs	60
Momentum	0.9
Activation function	ReLU
Learn rate drop factor	0.0
Learn rate drop period	0.0

**Table 6:** Different layer ICN models.

Layers	Training Loss	Training Accuracy	Validation Loss	Validation Accuracy	Execution Time
6	0	100%	0	96%	6 min 47 s
7	0	100%	0.2	96%	8 min 35 s
8	0.00033	100%	1.13	84%	17 min 18 s
9	0.18	97.66%	0.46	88%	35 min 34 s

**Table 7:** Pre-trained models.

Model	Training Loss	Training Accuracy	Validation Loss	Validation Accuracy	Execution Time
SqueezeNet	0.043	99.22%	0.037	100%	12 min 45 s
GoogleNet	0.003	100%	0.06	98.41%	28 min 28 s
ShuffleNet	0.003	100%	0.08	100%	23 min 23 s

**Table 8:** Defects classification using proposed seven-layered transfer learned ICNM.

Class	TPR	FNR	PPV	FDR	Training Loss	Training Accuracy	Validation Loss	Validation Accuracy	Testing Accuracy	Execution Time
Bird drop	100	0	100	0	0	100%	1.97 × 10^−5^	100%	97.62%	8 min 10 s
Patchwork	50	50	100	0						
Single	100	0	100	0						
String	100	0	100	0						
Block	100	0	94.4	5.6						

**Table 9:** Defects classification using pre-trained networks.

Network	Class	TPR	FNR	PPV	FDR	Training Loss	Training Accuracy	Validation Loss	Validation Accuracy	Testing Accuracy	Execution Time
Squeeze Net	Bird drop	100	0	100	0	0.009	100%	0.24	94.12%	100%	12 min
	Patchwork	100	0	100	0						
	Single	100	0	100	0						
	String	100	0	100	0						
	Block	100	0	100	0						
Google Net	Bird drop	100	0	100	0	0.013	100%	0.07	97.62%	97.62%	28 min 21 s
	Patchwork	50	50	100	0						
	Single	100	0	94.1	5.9						
	String	100	0	100	0						
	Block	100	0	100	0						
Shuffle Net	Bird drop	100	0	100	0	0.003	100%	0.2	94.12%	97.62%	21 min 38 s
	Patchwork	100	0	100	0						
	Single	100	0	94.1	5.9						
	String	80	20	100	0						
	Block	100	0	100	0						

**Table 10:** Augmented data-based defects classification.

Network	Class	TPR	FNR	PPV	FDR	Training Loss	Training Accuracy	Validation Loss	Validation Accuracy	Execution Time
Seven-layered transfer learned model	Bird drop	100	0	100	0	7.02 × 10^−5^	100%	1.06	93.3%	11 min 1 s
	Patchwork	100	0	91.7	8.3					
	Single	100	0	91.7	8.3					
	String	90.9	9.1	100	0					
	Block	90.9	9.1	100	0					
GoogleNet	Bird drop	90.9	9.1	100	0	0.017	99.22%	0.43	92.7%	40 min 20 s
	Patchwork	100	0	100	0					
	Single	100	0	84.6	15.4					
	String	100	0	100	0					
	Block	90.9	9.1	100	0					
ShuffleNet	Bird drop	100	0	100	0	0.0028	100%	0.27	91.1%	31 min 19 s
	Patchwork	100	0	100	0					
	Single	100	0	91.7	8.3					
	String	100	0	100	0					
	Block	90.9	9.1	100	0					
Squeeze Net	Bird drop	100	0	100	0	0.00085	100%	1.17	89.9%	17 min 56 s
	Patchwork	100	0	100	0					
	Single	100	0	73.3	26.7					
	String	90.9	9.1	100	0					
	Block	72.7	27.3	100	0					

## Data Availability

The data presented in this study are available on request from the corresponding authors.
